# Early direct competition does not determine the community structure in a desert riparian forest

**DOI:** 10.1038/s41598-018-22864-y

**Published:** 2018-03-14

**Authors:** Guilin Wu, Shaowei Jiang, Hui Liu, Shidan Zhu, Duoduo Zhou, Ying Zhang, Qi Luo, Jun Li

**Affiliations:** 1 State Key Laboratory of Desert and Oasis Ecology, Xinjiang Institute of Ecology and Geography, Chinese Academy of Sciences, Urumqi, Xinjiang China; 2 Key Laboratory of Vegetation Restoration and Management of Degraded Ecosystems, South China Botanical Garden, Chinese Academy of Sciences, Xingke Road 723, Tianhe District, Guangzhou, 510650 PR China; 3 University of Chinese Academy of Sciences, Yuquan road 19A, Beijing, 100049 China; 4 Guangdong Provincial Key Laboratory of Applied Botany, South China Botanical Garden, Chinese Academy of Sciences, Xingke Road 723, Guangzhou, 510650 China; 5 Guangxi Key Laboratory of Forest Ecology and Conservation, College of Forestry, Guangxi University, Nanning, Guangxi 530004 China

## Abstract

In riparian zones along the Tarim River in northeastern China, the co-dominance by *Populus euphratica* and *Tamarix ramosissima* at the early succession stage shifts to *P. euphratica* dominance in the late stages. However, little is known about how this shift is mediated by the highly variable water conditions in riparian zones. Here we conducted a mesocosm experiment in which we measured the physiological and morphological traits of these two co-occuring species grown in mixtures under simulated favorable groundwater condition and no groundwater availability. Results indicated that *T. ramosissima*, in comparison to *P. euphratica*, had much lower *WUE*, less proportion of root biomass under favorable groundwater condition. Under no groundwater condition, *T. ramosissima* also showed higher maximal quantum yield of PSII which allowed it to accumulate higher aboveground and total biomass. Therefore, regardless of groundwater conditions, *T. ramosissima* exhibited superior competitive advantages against *P. euphratica* under direct competition condition, which demonstrates that the dominance shift was not resulted from the direct competition at seedling stage. Our findings further imply that a strategy of “sit and wait” in *P. euphratica* might favor its growth and survival when suffered flooding disturbances, thus allowing *P. euphratica* not being excluded through competition at early successional stage.

## Introduction

Environmental variability is often considered as an important influence on community structure because of its effects on population growth and species interactions such as competition. In particular, temporal environmental variability is commonly believed to promote species diversity by preventing competitive exclusion that would otherwise occur^1,2^. In theory, given a constant environment, species with superior advantage would drive out the weaker ones. When the environment is variable, one species might not always have superior advantage over others, thus results in coexistence^3–5^. Study the competition effects under various environment, not only helps us to explain how species replacement and community succession, but also provides guidelines for forest management and reconstruction^5,6^.

Riparian plants are subject to high variability in water availability due to hydrological fluctuations^7–10^. The spatial-temporal variations of water availability, interacted with plant-plant competition, can further determine the riparian plant distribution^11^. Because competition takes place in the context of environmental conditions, competitive advantages need to be considered over a range of conditions^12^. In desert riparian environment, for example, species with the ability to grow rapidly in wet years and tolerate water deficit in drought years may be more competitive^13^.

Tarim River is a 1321-km-long inland river, located in the arid region of northwestern China. Desert riparian forest occurs along the river, dominated by a tree species *Populus euphratica* Oliv.^14^. However, at the seedling stage near the active river channel, there are seedlings of another species, *T. ramosissima*, co-establish and co-dominate the riparian community^15^. The shift from the dominance of both species at the seedling stage to the dominance of only *P. euphratica* at late successional stage suggests that *P. euphratica* out-competes *T. ramosissima* during the succession. Because *P. euphratica* is a tree species while *T. ramosissima* is a shrub species, earlier occupancy of the riparian space to avoid being suppressed by the other species would win a competitive advantage, so that early competition is critical for the riparian community succession.

Previous studies showed that competition during the succession is mediated by environment variability. Implications from the southwestern USA riparian ecosystems where *Tamarix* have replaced native popular and willow species^16^ indicate that the dominance of *Tamarix* was largely resulted from reduced competition intensity by native species due to groundwater decline and/or flood disappearance^17^. Another example was that *Synedra* was competitively advantage over *Fragilaria* in both constant and varying cultures, but the rate of competitive exclusion was slower in varying cultures^18^. As for the two species in the Tarim River riparian community, seedlings of *T. ramosissima* exhibits competitive advantages over seedlings of *P. euphratica* even under favorable water environment where *P. euphratica* was most likely expected to out compete *T. ramosissima*^19^. *T. ramosissima* was also more drought tolerant^20^ and able to accumulate more biomass under variable groundwater conditions in monocultures^15^. These studies all suggested that *T. ramosissima* was more competitive than *P. euphratica*, contrary to the dominant position of *P. euphratica* in the forests along the Tarim River. Thus further work on plant interactions between the two species under variable water environment is needed.

Since the growth of both species are highly depended on groundwater availability in Tarim River Basin^21^, here we examined the competition outcome between these two co-occurring species under simulated groundwater-available condition (the groundwater was shallow but no plants were inundated) and groundwater-unavailable environment (the groundwater is not available for seedlings). Under each condition, *P. euphratica* and *T. ramosissima* seedlings were grown in mixtures and ecophysiological traits relative to competitive ability were measured, including leaf photosynthesis, biomass allocation and root distribution. We asked: (1) whether competition between the two species was mediated by groundwater conditions, and then (2) whether the direct competition at the early stage is responsible for the shift of dominant species during the community succession in the fields of the Tarim desert riparian forests.

## Results

### Plant water status

**Figure 1:**
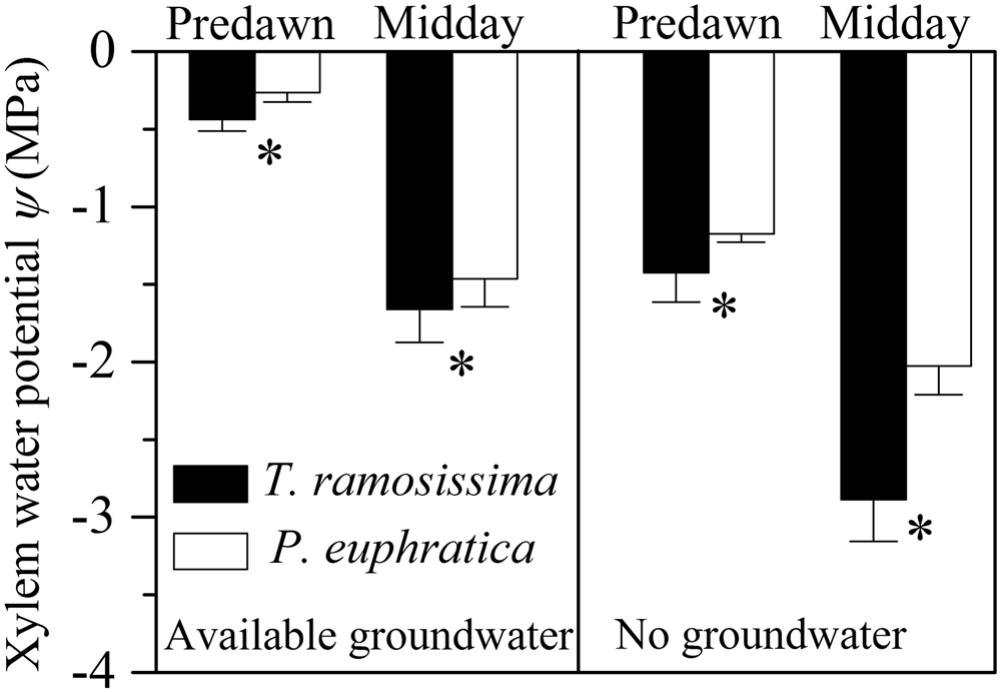
Predawn (*ψ*_*pd*_) and (*ψ*_*md*_) midday xylem water potential for *P. euphratica* and *T. ramosissima* under available groundwater and no groundwater environment. Values are mean ± SD (n = 3). Asterisk indicates significant difference with paired bars (* represents *P* < 0.05, NS represents no significance, the same below).

*ψ*_*pd*_ and *ψ*_*md*_ in *P. euphratica* were significantly higher (*P* < 0.05) than those of *T. ramosissima* regardless of water environments (Fig. 1).

### Leaf gas exchange

**Figure 2:**
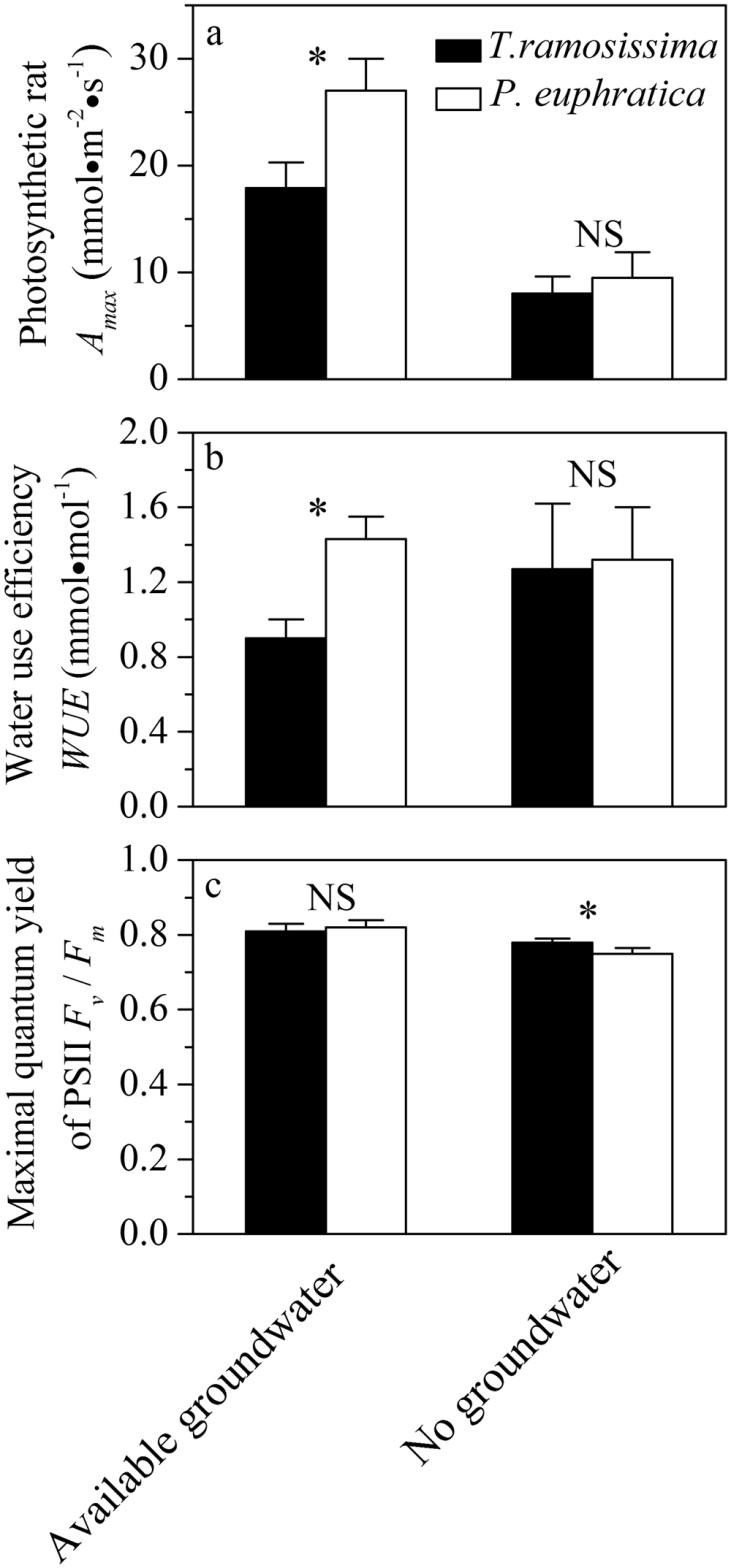
Photosynthetic rate (*A*_*max*_), water use efficiency (*WUE*), Maximal quantum yield of PSII (*F*_*v*_/*F*_*m*_) for *P. euphratica* and *T. ramosissima* under available groundwater and no groundwater environment. Values are mean ± SD (n = 3).

Maximum net photosynthesis rate (*A*_*max*_) and water use efficiency (*WUE*) were significantly higher (*P* < 0.05) in *P. euphratica* than *T. ramosissima* under available groundwater condition, but there was no significant difference between the two species under no groundwater environment (Fig. 2a,b). Maximal quantum yield of PSII (*F*_*v*_/*F*_*m*_) was significantly higher (*P* < 0.05) in *T. ramosissima* under no groundwater condition, while there was no significant difference under available groundwater environment (Fig. 2c).

### Biomass allocation and root distribution

**Figure 3:**
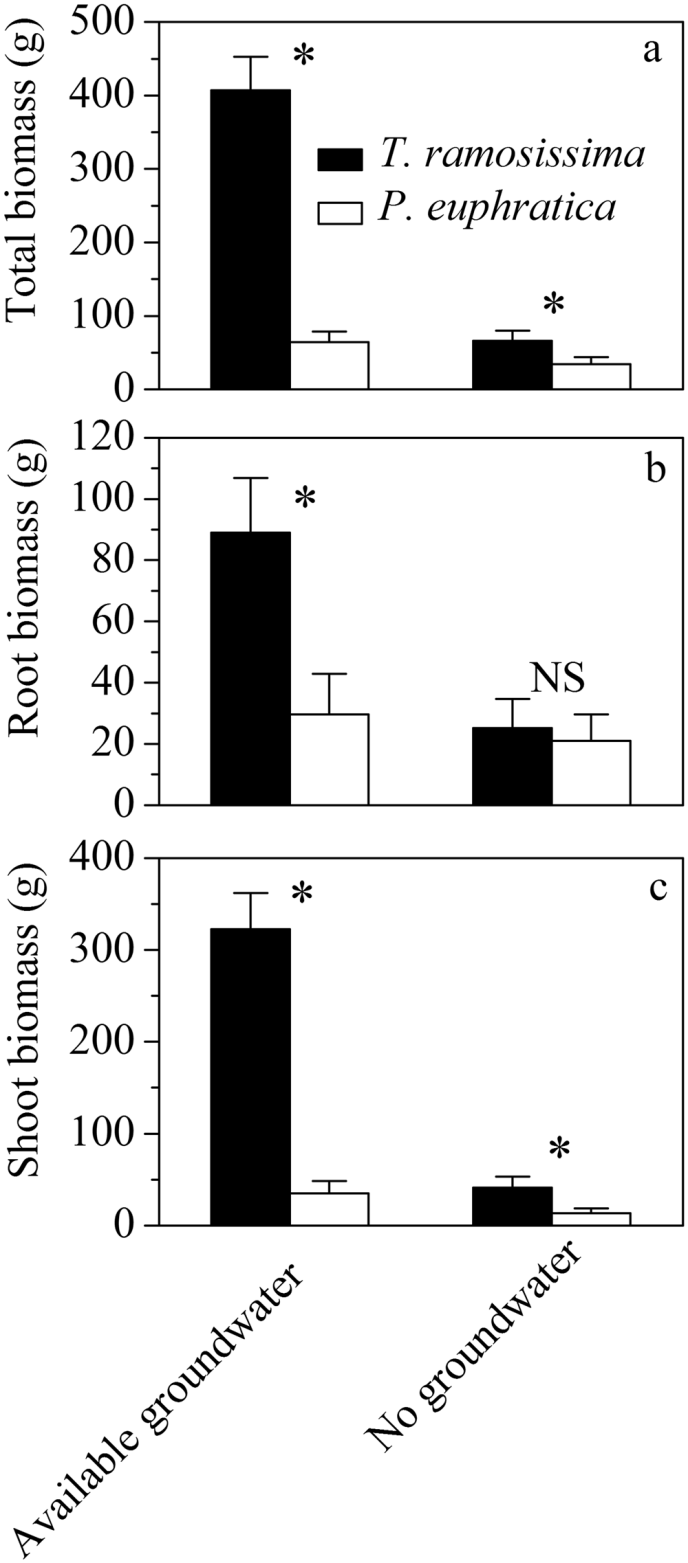
Total, root and shoot biomass for *P. euphratica* and *T. ramosissima* under available groundwater and no groundwater environment. Values are mean ± SD (n = 3).

Total biomass and aboveground biomass were significantly higher (*P* < 0.05) in *T. ramosissima* than that of *P. euphratica* regardless of water environments (Fig. 3a,b). Root biomass was significantly higher (*P* < 0.05) in *T. ramosissima* than *P. euphratica* under available groundwater environment, while there was no significant difference between the two species under no groundwater condition (Fig. 3c).

**Figure 4:**
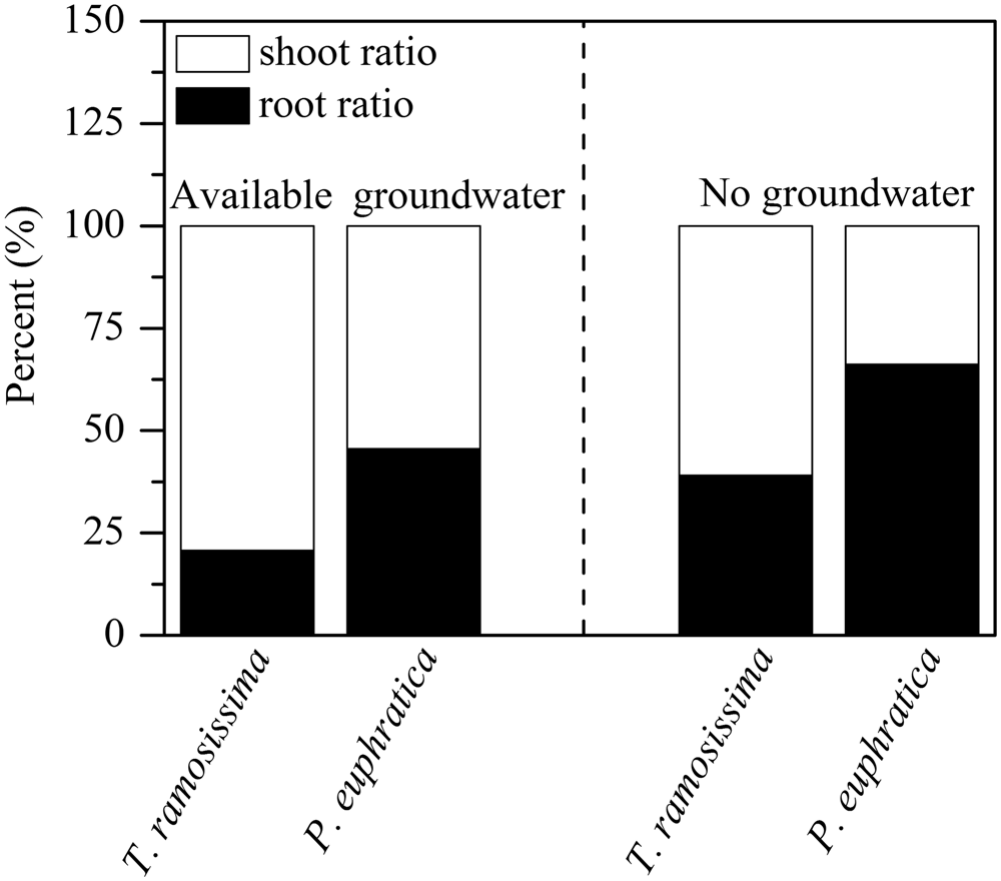
Root and shoot ratio for *P. euphratica* and *T. ramosissima* under available groundwater and no groundwater environment. Values are mean ± SD (n = 3).

The percentage of root biomass in *P. euphratica* was significantly higher (*P* < 0.05) than *T. ramosissima*, shoot biomass in *P. euphratica* was significantly lower (*P* < 0.05) than *T. ramosissima* correspondingly regardless of water condition (Fig. 4).

**Figure 5:**
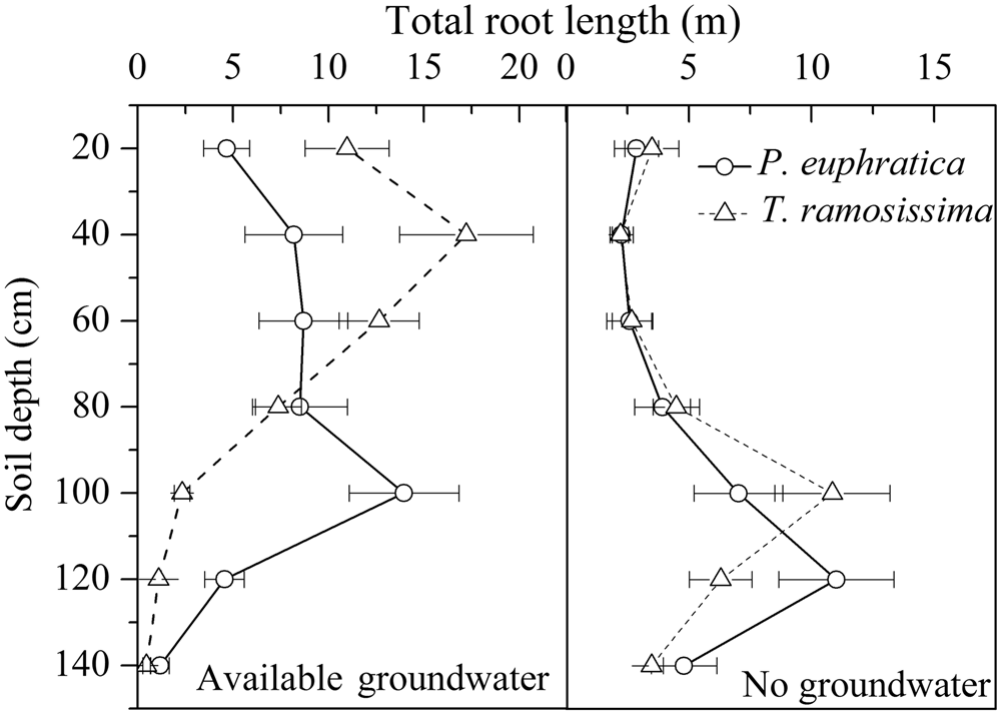
Total root length at different soil depths for *P. euphratica* and *T. ramosissima* under available groundwater and no groundwater environment. Values are mean ± SD (n = 3).

There was significant difference in total root length between the two species. Under available groundwater condition, with the increasing of soil depth, total root length for *T. ramosissima* increased at first, then decreased, it reached the maximum at 40 cm depth. While root length of *P. euphratica* increased firstly, then stayed constant from 40 cm to 80 cm, reached the peak at 100 cm depth. Under no groundwater condition, root length of *T. ramosissima* peaked at 100 cm depth, while *P. euphratica* peaked at 120 cm depth (Fig. 5).

**Figure 6:**
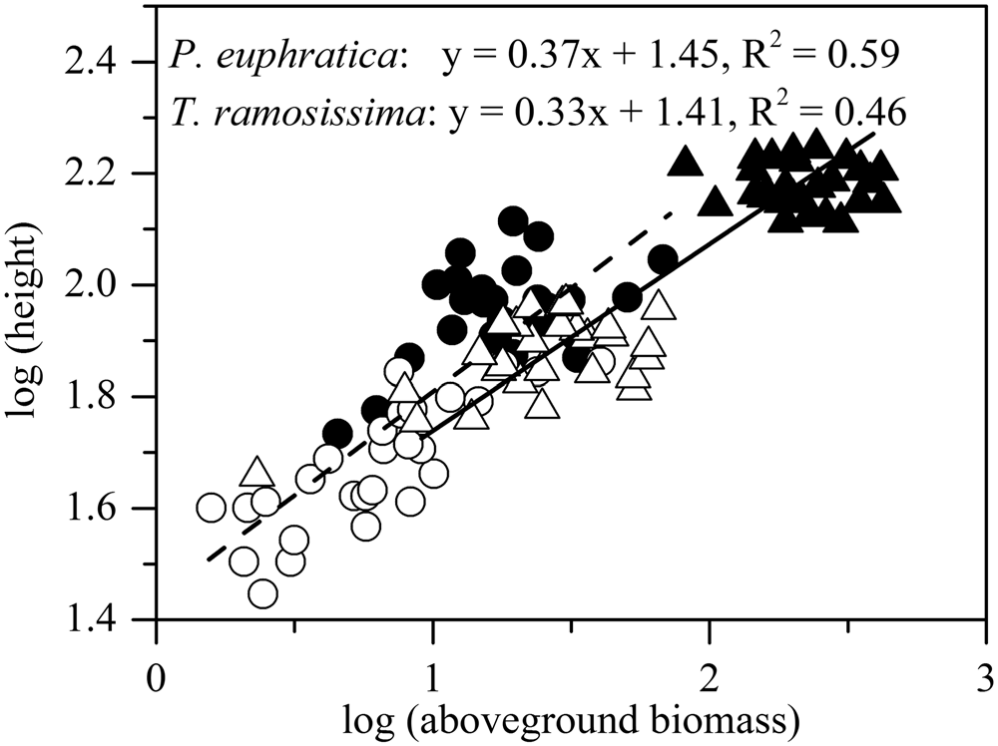
Relationship between log (biomass) and log (height) for *P. euphratica* and *T. ramosissima* under available groundwater and no groundwater environment. Data of *P. euphratica* under no (open circles) and high (filled circles) groundwater conditions were modeled with a dashed line, while *T. ramosissima* under no (open triangle) and high (filled triangle) groundwater conditions were modeled with a solid line.

Both species showed positive relationships between log (height) and log (aboveground biomass). Height of *P. euphratica* increased with aboveground biomass quicker (higher regression slope) than *T. ramosissima* (Fig. 6).

## Discussion

Our results indicated that *T. ramosissima* had competitive advantages over *P. euphratica*, evidenced by higher aboveground as well as total biomass regardless of groundwater conditions. The consistent results under two groundwater conditions suggested that the dominance shift from early to late successional stages in Tarim desert riparian forests was not resulted from early direct competition. Furthermore, we found that the competition outcome was associated with the growth strategies in the studied species at the early stage. *P. euphratica* allocated higher proportional biomass to roots under both groundwater conditions, a strategy favoring survival rather than growth in riparian environments. On contrary, the relationships between plant height and biomass suggested that *T. ramosissima* tends to occupy horizontal space, a strategy as weeds^22,23^ that suppress its competitors through rapid growth.

Ecophysiological traits of plants reflect adaption strategies to various environments, which provide guidelines for species replacement during forest community succession^24,25^. In agreement with Wu *et al*.^19^, in our study, *T. ramosissima* had superior advantages over *P. euphratica* under available groundwater condition (Figs 1 and 3). *T. ramosissima* accumulated more biomass in two ways. Firstly, the significantly lower *WUE* suggested that *T. ramosissima* accumulated carbon at high expense of water (Fig. 2), which was in agreement with Brotherson & Field^26^. Secondly, although *T. ramosissima* had lower net photosynthetic rate, and much lower percent of root biomass allocation in *T. ramosissima* (Fig. 4) indicated that it invested more to aboveground biomass with higher potential to increase leave areas, thus advantaged more CO_2_ accumulation. Li *et al*.^15^ demonstrated that *T. ramosissima* had significant higher percent leaf biomass than *P. euphratica* under various groundwater conditions. Benefited from these traits, *T. ramosissima* could suppress *P. euphratica* under available groundwater condition.

Under no groundwater environment, *T. ramosissima* showed more negative *Ψ*_*md*_ than *P. euphratica* (−2.9 MPa for *T. ramosissima* versus −2.0 MPa for *P. euphratica*, Fig. 1). *T. ramosissima* also has a more negative water potential inducing 50% loss of hydraulic conductivity (*P*_50_) (−4.5 MPa for *T. ramosissima* and −0.70 MPa for *P. euphratica*)^20,27^, thus according to the estimation of hydraulic safety margin by Choat *et al*.^28^, it is clear that *P. euphratica* was in a higher risk of hydraulic failure. Furthermore, significantly lower maximal quantum yield of PSII *F*_*v*_/*F*_*m*_ of *P. euphratica* (Fig. 2) also demonstrated the higher degree of water stress. Under no groundwater condition, both species had similar *WUE*, the higher drought tolerance ensured *T. ramosissima* to accumulate more biomass (Fig. 3). Overall, our results demonstrated that stronger competitiveness of *T. ramosissima* in our experiment benefited from its water waste strategy and higher aboveground biomass allocation under available groundwater condition, and more drought tolerance under no ground water condition.

Water availability in riparian zone in arid and semi-arid region was highly variable due to hydrological fluctuations^10,29^. Thus, root allocation strategy in such environment is critical for the survival and growth in species. Higher proportion of roots allocation and deeper roots distribution was advantage survival of species when water is unfavorable water environment, but would restrict plant growth under favorable water environments. Less proportion of root allocation and shallower roots distribution would advantage growth of species when water is favorable, but might have water stress risk when water environment is unfavorable^7^. In our study, in agreement with Busch and Smith^30^, Horton and Clark^31^, both species were phreatophytic, their roots nearly attached the bottom of the pools either in favorable or unfavorable water condition, but *P. euphratica* had higher roots biomass allocation and deeper root length distribution than that of *T. ramosissima* regardless of groundwater (Fig. 5), suggested advantage in growth for *T. ramosissima* under available groundwater condition and in survival for *P. euphratica* under no groundwater environment.

As a light-demanding species, *P. euphratica* should grow higher to avoid being shaded by *T. ramosissima*. Yokozawa and Hara^32^ indicated that allometric relationship between plant height and aboveground mass implied competitive strategies for light. Taller plants will are able to project their leaves in the highest positions of the canopy where they receive the more light intensities^33,34^. On the other hand, there are costs related to increase height: plants invest a disproportionate amount of biomass in support tissue. In our study, to access more light intensities, *P. euphratica* tended to increase height at the cost of lateral growth (Fig. 6). Such strategy was advantage to the survival of *P. euphratica*. But *T. ramosissima* tended to occupy horizontal space to suppress *P. euphratica*.

Based on the root allocation, relationship between height and biomass in *P. euphratica*, it is possible that it could “sit and wait” until conditions become favorable. For instance, *T. ramosissima* suffers from large percentage of biomass loss due to extreme stress or disturbance, thus releasing the intensity of competition between them. This is a reasonable hypothesis to explain the domination of *P. euphratica*. As desert riparian zone is highly disturbance by runoff of river, seedlings near channel were more vulnerable. Plants with higher aboveground biomass and horizontal growth have higher risk of being uprooted by extreme floods^35^. So plants invest more to aboveground biomass have higher risk to loss more in desert riparian zone, while the strategy in *P. euphratica* might make it suffer less loss from disturbance. Moreover, *P. euphratica* have lancet-like leaves at seedling stage, and such leaf shape is known to decrease flush resistance, thus favoring decreasing aboveground biomass loss during floods^35^. As suggested by the “storage effect” theory^4^, *P. euphratica* might “store” more underground biomass in various groundwater environments, which buffered the effects of disturbance. All the above competition strategies explained the co-existence of both species at the seedling stage. As long as *P. euphratica* can survive the early stage (as we observed that the mortality in *P. euphratica* seedlings was negligible although they were obviously suppressed by *T. ramosissima*), it will out compete *T. ramosissima* in the late stage of the plant community in the Tarim riparian zones.

Our study implied that the success of *P. euphratica* in Tarim riparian zone probably rely on disturbance that change or weaken competition direction in *T. ramosissima*. Shafroth *et al*.^36^ indicated that dam construction in the western USA altered disturbance thus accelerated the invasion of *Tamarix*. With more water consuming and manual control of runoff with the development of agriculture in Tarim region, the decrease of water volume and natural flow in Tarim River will be serious ecological problem^37^. The larger distribution area of *T. ramosissima* than that of *P. euphratica*^38^ suggested the changing of hydrological environment in Tarim region, which might lead to the expansion of *T. ramosissima*. Therefore, management of natural flow in Tarim River is critical for final community structure in Tarim region, and for the maintenance of *P. euphratica* forest.

Our study demonstrate that *T. ramosissima* has overwhelming competition advantages at the seedling stage over *P. euphratica* regardless of groundwater conditions, so that the early direct competition between the two species is not the reason for the dominance of *P. euphratica* in late successional stages in the Tarim River Basin. In details, seedlings of *P. eupharitca* allocated more proportional biomass to root, and tended to grow higher at the cost of lateral growth when suppressed by *T. ramosissima*. Such a strategy allows *P. euphratica* to survive at the early successional stage. A strategy of “sit and wait” in *P. euphratica* might favor its growth and survival when suffered flooding disturbances, which could release the intensity of direct competition at the early successional stage, leading to the dominance by *P. euphratica* in the riparian plant communities in fields. Therefore, it is critical to maintain natural flooding regimes for the management of the desert riparian forest in the Tarim Basin.

## Materials and Methods

### Study sites

Experiments were carried out at the Aksu Water Balance Station, Chinese Academy of Sciences (40°27′N, 80°45′E, hereafter Aksu Station), located in the Tarim Basin, northwestern China. The region is characterized by a hyperarid climate, with an annual mean precipitation of 45.7 mm but an annual mean potential evaporation greater than 2500 mm. In August 2011, seeds of both species were collected from natural populations and then sown in a common garden in the Aksu Station. Then in March 2013, seedlings with heights ranging from 35 to 42 cm were transplanted into outdoor concrete pools designed for simulating different groundwater conditions.

### Experimental design

The experimental pools, 5 m^2^ of each in size, were filled with soil collected from riparian zones of the Tarim River, with drainage valves at different depths to control groundwater depth. Details of the experiment design were described by Wu *et al*.^19^. Briefly, seedlings of both species were grown alternatively at a space of 0.4 m * 0.4 m, total 28 seedlings in each pool that was similar in density to field communities at this stage. There were 6 pools in total, three for favorable groundwater treatment, and the other 3 for no groundwater available. For the available groundwater treatment, a drainage valve at 0.4 m depth in each pool was maintained open, and flooding irrigations were carried out weekly during the experiment, through which the groundwater was maintained within a range between 0.4 and 0.6 m below soil surface. For no groundwater treatment, irrigation was ceased after middle July when the seedlings were successfully established in pools. For the extremely low precipitation and great evaporative demand in Tarim region, rainfall was not excluded for the no groundwater treatment.

### Data collections

We determined plant water status by measuring leaf water potential at predawn (*Ψ*_*pd*_) and midday (*Ψ*_*md*_) with a pressure chamber (PMS, Corvallis, OR, USA). Measurements were carried out between 06:30 and 07:30 for *Ψ*_*pd*_ and between 15:30–16:30 for *Ψ*_*md*_. A minimum of nine fully expanded mature leaves from three individuals per species in each pool were sampled for leaf water potential measurements. Water status for *P. euphratica* and *T. ramosissima* were measured three times from July to September.

Leaf gas exchange was measured between 10:30 and 12:30 using a portable photosynthesis system equipped with a CO_2_ injector (Li6400, Li-Cor, Lincoln, USA). Based on preliminary trials, the photosynthetic photon flux density was set at 1500 μmol m^−2^s^−1^ to ensure that light-saturated photosynthesis rates were reached for the two study species. Ambient CO_2_ was maintained at 400 μmol mol^−1^. Similar to the sampling way for leaf water potential measurements, a minimum of nine fully expanded mature leaves from three individuals per species in each pool were selected, for the quantification of maximum net CO_2_ assimilation rate (*A*_*max*_), stomatal conductance (*g*_*s*_) and transpiration rate (*T*_*r*_), leaf water use efficiency (*WUE*) was calculated as *A*_*max*_/*T*_*r*_. Chlorophyll fluorescence parameters (*F*_*v*_/*F*_*m*_) were measured in a standard fluorescence leaf chamber with a Li-6400 portable photosynthesis system. Prior to the measurement in the early morning, a clip was placed on each leaf for 30–40 min for dark adaptation^39^. Gas exchange for *P. euphratica* and *T. ramosissima* were also measured three times from July to September.

At the end of the experiment, nine seedlings of each species for each treatment were harvested to measure aboveground biomass. We dug a ditch around the pool thus made an earth cube in the center of the pool. Then the cube was soaked with water for a few hours to facilitate removal of roots from the loamy soils with a spray nozzle. We used tape to measure root length at each layer for the selected individuals. All biomass was dried in an oven at 65 °C for 72 h and then weighed.

### Data analysis

We use *t*-test to analysis xylem water potential, above-, below-biomass, *A*_*max*_, *WUE*, *F*_*v*_/*F*_*m*_ between species in each water treatment. We fitted the relationship between log-transformed height and aboveground biomass with linear models. All statistical tests were performed using SPSS 13.0 (SPSS Inc., Chicago, IL, USA).
